# Improvement and implementation of central sterile supply department training program based on action research

**DOI:** 10.1186/s12912-024-01809-z

**Published:** 2024-03-18

**Authors:** Ting Hu, Juanli Huang, Sixing Jiang, Ruixue Hu, Yongdeng Huang, Wei Pan, Liangying Yi

**Affiliations:** 1 Department of Sterile Processing Nursing, West China Second University Hospital, Sichuan University, Chengdu, Sichuan China; 2 Key Laboratory of Birth Defects and Related Diseases of Women and Children (Sichuan University), Ministry of Education, Chengdu, Sichuan China

**Keywords:** The central sterile supply department, Nurses, Experience, Ethics education

## Abstract

**Background:**

This study aimed to improve and implement the central sterile supply department (CSSD) training program through action research and to evaluate its effect.

**Materials and methods:**

The project was underpinned by action research. The problems that occurred in training were identified following scenario analysis, interviews, group discussions, and expert consultation to develop improvement measures. The training program characterized by CSSD was developed in the spiral circular process of “Plan-Act-Observe-Reflect”.

**Results:**

After the two rounds of training, the CSSD nurses significantly improved their professional knowledge, skills, and satisfaction with training compared with those before improvement. The nurses’ overall satisfaction with the training and their performance improved.

**Conclusions:**

The implementation of the training program designed based on action research can improve CSSD nurses’ professional knowledge and skills and increase their enthusiasm for learning, laying the foundation for CSSD talent development.

## Background

The Central Sterile Supply Department (CSSD) is a hospital department responsible for the cleaning, disinfection, sterilization and supply of sterile articles within the hospital [1]. CSSDs in China are generally directly managed by nursing departments, with some are managed by the Department of Nosocomial Infection Prevention and Control. The sterile supply discipline occurred later than other nursing subjects. Many newly recruited CSSD nurses have limited knowledge of CSSD and its development prospects [2]. The training for CSSD nurses has lagged behind that of clinical nurses [3]. CSSD is one of the key units involving in nosocomial infection prevention and control. The quality of sterile articles directly affects medical quality and patient safety [4]. Professional CSSD knowledge involves a wide range of subjects, including physics, biology, and chemistry. As nursing program at schools do not usually provide knowledge and skills in sterile supply, on-the-job training for CSSD staff is necessary to build a professional CSSD team and promote CSSD development. The National Nursing Development Plan of China repeatedly emphasizes the significance of the development and on-the-job training of specialized nurses [5]. Adult learning is generally based on utility. In adult learning, learners care about whether the study content is closely related to the problems they encounter in their daily work [6], and want to obtain the knowledge and skills that can be applied in their work, to enable them to work better. Adults have certain social experiences, imagination, analytical ability, and a strong acceptance ability. Because adults are generally busy earning a living, it is challenge leaving their jobs to pursue study opportunities [7]. Therefore, how to efficiently develop on-the-job training is a problem that needs to be addressed urgently for CSSD management. Action research is an educational research method widely employed in Western countries since the 1970s [8]. In the nursing field, action research was introduced and extensively applied in the 1990s [9]. Action research involves integrating research with the resolution of practical workplace issues, constituting a small-scale intervention in the real-world activities, and carefully examining the impacts of such intervention [10]. Action research is exploratory work through collaborative self-reflection to solve practical problems [11]. It is a spiral process consisting of four steps: “Plan-Act-Observe-Reflect” [12]. Action research emphasizes the equal status of researchers and participants, fostering a collaborative relationship without hierarchy or authority. Both parties engage in open dialogue and communication throughout the research process. In this study, the action research approach is employed to refine and optimize the training system. It can greatly ensure that the improved training is in line with the needs of the trainees and is trainee-oriented, not developed unilaterally by the trainers or managerial personnel. This study aimed to use the action research method to perfect the training system and improve the learning effect, to improve CSSD staff’s job satisfaction.

## Materials and methods

### Research participant

The CSSD nurses who were working in a Grade A tertiary women’s and children’s hospital in Sichuan Province, China were invited to participate in this study. The inclusion criteria were as follows: (1) nurses who were working in the CSSD; (2) who were working under the formal employment contract signed with the hospital or the state-guaranteed lifetime employment. The exclusion criteria were as follows: (1) who had been absent from work for ≥ 3 months; (2) who would retire within 1 year; and (3) age > 50. A total of 40 nurses were included. The demographic information of the included nurses is presented in Table 1.

**Table 1:** Demographic information of CSSD nurses (*n* = 40)

	Item	Number (*n*)	Percentage (%)
Gender			
	Male	4	10
	Female	36	90
Age			
	18–25	1	2.5
	26–30	28	70
	31–40	8	20
	41–50	3	7.5
Educational attainment			
	Junior college diploma	2	5
	Undergraduate qualification	35	87.5
	Master’s degree	3	7.5
Working years			
	1–3 years	12	30
	4–6 years	15	37.5
	7–10 years	10	25
	> 10 years	3	7.5
Professional title			
	Nurse Practitioner	28	70
	Supervising nurse	10	25
	Associate senior nurse	2	5

### Establishment of a research team

The research team consisted of the authors, 1 department-level head nurse, 2 other head nurses, and 4 instructors with more than 5 years of teaching and training experience. The research team revised the training program content, implemented the training, and assessed learning effects of the nurses who participated in the training. The research team also designed the training assessment plan and the unified assessment criteria in advance. The 40 CSSD nurses who were the members of the action research team, received the training, and provided feedback on the problems during the training.

### Action research steps

The first step in adopting action research is to identify the problem. We identify the issues that arose during the training process. Following the two spiral processes of “Plan-Act-Observe-Reflect,” the CSSD hierarchical training program was perfected to improve the learning effect. The specific methods are as follows:

### Identify the problems

Based on training satisfaction feedback, training quality control, personnel interviews, and the theoretical and skill assessment results after the hierarchical training in 2022, the following problems were identified: (1) low level of training satisfaction; (2) the assessment content was not fully targeted; (3) the nurses did not firmly grasp theoretical knowledge; (4) CSSD work was not conducted in conformity with guidelines; and (5) the training content did not show obvious progression in knowledge and learning. After semi-structured interviews were conducted with the 40 nurses, the following problems were identified: (1) the traditional training methods decreased the nurses’ interest in learning; (2) the repeated training content did not show obvious progression in knowledge and learning; and (3) the training lasted for a long time. The interviewees generally felt that the training was very important and was one of the important means of developing CSSD talents. They hoped to receive systematic and practical training to help them better deal with work and career growth. The above-mentioned problems provided a basis for improving the training program.

### Develop a plan

Improvement strategies and plans were developed based on scenario analysis, group discussions, literature review, and expert consultation. Scenario analysis involves examining factors influencing the implementation of training and the resistance faced by nurses in the CSSD during their actual work scenarios. The goal is to identify ways to minimize the impact factors and reduce resistance to training in this specific context. Before engaging in group discussions, relevant improvement solutions and strategies were gathered through a comprehensive literature review. Additionally, the research team consulted industry experts to assess the completeness and feasibility of these proposed solutions and strategies. Subsequently, the research team conducted brainstorming sessions and focused discussions to consolidate the findings. As a result, five key areas were identified for the final improvement plans and strategies:

1. Build a CSSD knowledge system, as shown in Table 2. A perfect knowledge system is a prerequisite for high-quality training and can help trainers better develop training program;
2. For training methods, various measures, such as face-to-face teaching, operation demonstration, workshops, simulation experiences, games, root cause analysis, and mind mapping, were used to target the delivery of relevant knowledge according to the characteristics of students. Meanwhile, online media-assisted teaching and encouraging students to participate in training could effectively improve trainees’ enthusiasm for leaning;
3. For assessment, a software application or public website could be used in the remote assessment and pre-test simulation stages. To ensure fairness in the assessment, the quarterly examinations were conducted offline. After the instructor who was the teaching director set the questions and the head nurses reviewed them, the exam test paper was issued to other instructors for assessment. The passing score was 60 points. The trainees who failed the exam needed to resit it until they passed it. Once they passed the resit exam, the result of the re-sit exam would be recorded as their result for the training;
4. Reasonably arrange the length of training sessions. The training was provided once a month and lasted 60–90 min per session. To promote the implementation of the training program and strengthen the learning atmosphere within the CSSD, a two-dimensional code concerning the CSSD training content was pasted on the CSSD’s bulletin board so that the CSSD nurses could scan it to study in their fragmented time;
5. Selection of training instructors. Instructors with rich work experience are an important strength in building a nursing team. They have acquired a high level of theoretical knowledge, skills, and work experience in nursing. Therefore, we selected the instructors who had more than 5 years of working experience, rich teaching and training experience, and were sterile supply experts. Because of the particularity of CSSD work which involves specialized equipment, we invited the engineers of the manufacturers for instruction. In addition, infection prevention and control and microbiology experts could also be invited for instruction to make full use of CSSD work resource.

**Table 2:** CSSD knowledge system

Zones	Steps	Training content	Training method	Assessment method	Expected results
1. Decontamination zone	1.1 Environmental requirements	Temperature, humidity, ventilation rate, and zone layout	Lecture	Theoretical exam	Score > 80
	1.2 Instrument collection	Collection criteria and tools for collection	Demonstration	Practical assessment	Score > 80
		Dress code requirements	Demonstration	Practical assessment	Score > 80
		Precautions for collection workflow, pre-processing, and transport	Scenario practice	Practical assessment	Score > 80
	1.3 Classification	Evaluate the risks of infections, instrument materials, and pollution levels	Demonstration	Practical assessment	Score > 80
	1.4 Check	Count the instruments, and check the functions of the instruments and the use of tracking system	Demonstration	Practical assessment	Score > 80
	1.5 Cleaning	Cleaning procedures for common and precise instruments	Demonstration	Practical assessment	Score > 80
		Cleaning procedures for instruments used by patients with specific infectious diseases	Scenario practice	Theoretical exam	Score > 80
		Cleaning medium: action principle and formulation of cleaning agent; requirements of cleaning water	Simulated experience	Theoretical exam	Score > 80
		Use, maintenance, and troubleshooting of cleaning equipment	Simulated experience	Theoretical exam	Score > 80
		Cleaning methods, including hand cleaning, mechanical cleaning, and ultrasonic cleaning	Demonstration	Practical assessment	Score > 80
	1.6 Disinfection	Physical disinfection and chemical disinfection	Lecture	Theoretical exam	Score > 80
	1.7 Drying	Hand drying and mechanical drying	Demonstration	Practical assessment	Score > 80
2. Packaging inspection zone	2.1 Environmental requirements	Temperature, humidity, ventilation rate, and zone layout	Lecture	Theoretical exam	Score > 80
	2.2 Inspection and maintenance	Cleaning quality, function inspection methods, and maintenance workflow of instruments	Demonstration	Practical assessment	Score > 80
	2.3 Packaging	Packaging workflow and precautions	Demonstration	Practical assessment	Score > 80
	2.4 Sterilization	Common sterilization methods and equipment use process and management	Mind mapping	Practical assessment	Score > 80
3. Sterile package storage zone	3.1 Environmental requirements	Temperature and humidity, ventilation rate, zone layout	Lecture	Theoretical exam	Score > 80
	3.2 Delivery	Inspection rules	Lecture	Theoretical exam	Score > 80
4. Monitoring zone	4.1 Quality monitoring	Different sterilization methods: physical, chemical, and biological monitoring methods, and precautions	Lecture	Theoretical exam	Score > 80
	4.2. Environmental and surface monitoring	Monitoring frequency, methods, and passing criteria	Lecture	Theoretical exam	Score > 80
	4.3 Hand hygiene	Indicators of hand hygiene, monitoring frequency, and passing indicators	Game	Theoretical exam	Score > 80

### Act to implement the plan

From June to December 2022, the theoretical knowledge and specialized skills were sorted out according to the revised knowledge system, and the training program was implemented according to the improved training method and the length of training sessions. The researchers participated in the development, implementation and summary of the training program, and organized group discussions to reflect on and summarize the monthly training. New problems encountered during the training were discussed to revise the training program content. The research team supervised each round of training for 3 consecutive months. Two rounds last for a total of 6 months.

### Observe and reflect

Observation is the investigation and record of the process and results of an action. It highlights the importance of action research in data collection. The researchers involved in the formulation, implementation, and evaluation of the training program, understood whether the training content was practical, communicated with the trainees to understand their participation, gave their opinions and suggestions on the content, length and method of the training, and provided training guidance. After the first round of training, the trainees’ satisfaction with the training content and method increased. During the supervision and guidance, the research team understood the trainees’ opinions and suggestions on CSSD training through semi-structured interviews and discussed and reflected with them. Semi-structured interviews involve informal discussions conducted based on a loosely outlined interview guide [13]. The research team developed an interview guide based on the implementation of the first training round. Face-to-face interviews were conducted with 40 participants, and the team made necessary adjustments during the interviews based on the actual circumstances. No specific requirements were imposed on the questioning style and order, the participants’ responses, the method of recording, or the time and location of the interviews. For example, interviews could take place after training or work hours, in the office, or in a work setting, creating a relaxed atmosphere for the interviewees. The research team summarized the problems observed and encountered in the training practice as follows: (1) CSSD works a 24-hour shift every day throughout the entire year. No matter when the training was conducted, some on-duty nurses were unable to participate in the training; (2) Despite having a comprehensive professional knowledge system, it was perceived as dry, leading to a decrease in nurses’ learning interest and low engagement in the training. (3) Strict assessments were conducted during the training, but the impact of the grades was not clearly reflected.

Based on the reflection and identification of issues following the initial observation, we made modifications and additions to the improvement plan for the first round as follows: (1) In the first round of action, the training sessions were relatively lengthy, posing challenges for on-duty nurses with busy clinical schedules to participate fully. In response, for the second round, we modified the approach by breaking down the content and restricting the explanation time for key topics to within 30 min. We aimed for concise and efficient training. Secondary key content was included in the department’s teaching resource library, accompanied by relevant videos. After self-study, spot assessments were conducted. (2) To enhance the training content, we expanded beyond the professional knowledge system. Additional topics included human resources management, medical quality and safety control, communication skills, office software knowledge, social skills, industry code of conduct, and knowledge related to party building. We also sought opinions from nurses at all levels, collecting input on subjects they wished to learn. This approach fully respected the learners’ preferences, resulting in diverse training content and increased interest in learning. (3) The purpose of assessments is to ensure a solid grasp of knowledge and regulate inappropriate behavior in the workplace. Therefore, we incorporated training assessment results as one of the indicators for selecting outstanding employees at the year-end evaluation. This approach serves as an incentive for nurses at all levels to take training assessments seriously and value their assessment performance.

### Evaluation method

The evaluation of the training program consisted of two components: (1) a theoretical exam on fundamental knowledge, specialized knowledge, rules and regulations, knowledge of nosocomial infection prevention and control, practice, team building, and management. The evaluation of theoretical performance is primarily conducted through monthly theoretical knowledge exams. The exam questions correspond to well-defined assessment criteria. Nurses undergo paper-based exams, and their actual scores are recorded. Each action cycle spans 6 months, and the final score is an average obtained by comparing scores before and after the cycle. For practical performance evaluation, nurses undergo a practical assessment, utilizing standardized operation scoring sheets such as the “Instrument Closed Wrapping Operation Standard.” Points are deducted for actions that do not meet the specified standards. The final score is an average obtained by comparing scores before and after the cycle. (2) operation skills. The trainees’ satisfaction with the length, content, method, materials and frequency of the training was investigated. The levels of satisfaction are divided into four categories: very satisfied; satisfied; basically satisfied; and dissatisfied. “Very satisfied” and “satisfied” are consider the satisfaction index [14].

### Statistical analysis

SPSS Statistics 25.0 was used for data processing. The enumeration data are expressed in frequency and percentage. The Chi-square test, pair testing, and variance analysis were conducted.

## Results

As shown in Table 3, Through the improvement of the training program, the nurses’ results in fundamental knowledge, specialized knowledge, rules and regulations, knowledge of nosocomial infection prevention and control, practice, team building, management, and operational skills improved. The overall score has increased from 68.10 ± 9.009 to 83.30 ± 8.576, with an increase of 17.8%. The operation skills score has increased from 86.43 ± 3.713 to 93.53 ± 3.508, with an increase of 7.5%. Table 4 shows the results of the improved training satisfaction survey. In general, the implementation of the improved training program resulted in greater than 28% increase in satisfaction for all nurses who participated in the training. Interestingly, nurses’ satisfaction with the length of the improved training increased the most, from 15 to 38, an increase of 60.5%. Nurses’ satisfaction with the training materials of the improved training program increased the least, from 25 to 35 points, an increase of only 28.5%. Nurses’ satisfaction with the improved training program’s training content, training course schedule, and training method increased by 46.9%, 37.5%, and 41.7%, respectively (*p* < 0.05).

**Table 3:** Results of assessment of theoretical knowledge and operation skills

Groups	Theoretical knowledge					Overall score	Operation skills
	Fundamental knowledge	Specialized knowledge	Rules and regulations	Nosocomial infection prevention and control	Practice, team building, and management knowledge		
1–6 months after action	14.20 ± 2.103	13.43 ± 2.510	13.43 ± 2.086	13.68 ± 2.005	13.38 ± 2.238	68.10 ± 9.009	86.43 ± 3.713
6–12 months after action	17.25 ± 1.918	16.73 ± 2.242	16.63 ± 2.059	16.08 ± 2.390	16.63 ± 2.072	83.30 ± 8.576	93.53 ± 3.508
T value	-10.736	-10.422	-10.172	-6.921	-8.954	-14.896	-12.597
*P* value	< 0.001	< 0.001	< 0.001	< 0.001	< 0.001	< 0.001	< 0.001

**Table 4:** Results of training satisfaction survey [n (%) *N* = 40]

Item	Before action	After action	χ^2^	*P*
Length of training sessions	15 (37.5)	38 (95)	29.5	< 0.001
Training content	17 (42.5)	32 (80)	11.8	0.001
Training methods	21 (52.5)	36 (90)	13.7	< 0.001
Training materials	25 (62.5)	35 (87.5)	6.6	0.01
Training schedule	25 (62.5)	40 (100)	18.4	< 0.001

## Discussion

By means of action research, it is possible to enhance the caliber of training in a dynamic manner, thereby fostering the advancement of CSSD nurses’ theoretical knowledge, operational skills, and satisfaction with the training. The theoretical results showed that the score of Fundamental knowledge increased from 14.20 ± 2.103 to 17.25 ± 1.918, and the score of Specialized knowledge increased from 13.43 ± 2.510 to 16.73 ± 2.242. The score for Rules and regulations increased from 13.43 ± 2.086 to 16.63 ± 2.059. The score for Nosocomial infection prevention and control increased from 13.68 ± 2.005 to 16.08 ± 2.390. Lastly, the scores for Practice, team building, and management knowledge increased from 13.38 ± 2.238 to 16.63 ± 2.072. Furthermore, the comparison of nurses’ pre-training and post-training scores clearly shows that the improved training methodology can effectively elevate the standard of nurses’ training.

The implementation of the optimized training program is helping to improve the training quality and the nurses’ learning enthusiasm. Action research is a research method that closely combines scientific research with problems arising in practical work [15]. Due to the rapid development of health care, CSSD needs to deal with more modern, precision instruments with complex structures. As an independent discipline, CSSD needs to continuously improve the training program [16] and develop a training mode with CSSD features. Continuous improvement of the weak points in work procedures can be used to improve the training program, promote the overall improvement of CSSD work, and gain more recognition of clinical departments so that the CSSD nurses can improve their professional identity and greatly increase their learning enthusiasm.

An optimized training program can improve training efficiency. we modified the approach by breaking down the content and restricting the explanation time for key topics to within 30 min. We aimed for concise and efficient training. Frequent or lengthy training sessions may increase nurses’ aversion to training and increase their job burnout [17]. Traditional training does not pay attention to the length of training sessions and the maximization of time and planning, resulting in a waste of fragmented time. Our training was based on fragmented time management [18], including the use of mobile learning tools (such as mobile phones and two-dimensional code on the CSSD’s bulletin board), which allowed the nurses to study in the fragmented time of their daily life and work. For the nurses to study together, the length of each training session is short, so they can maintain a high level of concentration and achieve better leaning effect. This can save the nurses’ time and improve training efficiency.

Action research can help nursing managers effectively improve training program. Nursing administrators participate with the researcher in the improvement of the training program during the research process. Action research is a circular process of continuous improvement through practice. In nursing, researchers who engage in continuous improvement of nursing quality use action research to conduct the research, give feedback on problems that occurred in the research, and constantly adjusted the plans, modes and methods of training. Continuous attention to the implementation process can improve the learning effect [12]. It is advisable to design the training plan according to the clinical needs and key points of quality control each month. If a cleaning problem is found to be the key source of quality control problems in a given month, it is necessary to adjust the training content in time to meet the needs of solving the cleaning problems. The wisdom and abilities of researchers and practitioners should be combined to solve problems together. Given the low enthusiasm for training, innovative training methods can be adopted, such as conducting root causes analyses of some frequently occurring problems through group discussions. The nurses can review the theoretical knowledge by identifying the root causes via brainstorming. Enhanced training programs can additionally enhance the efficacy of improving training programs, thereby aiding managers in gaining a deeper comprehension of nurses’ learning requirements and adapting training initiatives accordingly.

High-quality training is an important prerequisite for building a professional team. The CSSD’s development is critical to the development of the sterile supply discipline. The development of the sterile supply discipline is inseparable from high-quality technical and management personnel with a reasonable knowledge structure [19]. However, many CSSD managers in our country do not pay enough attention to this. Many CSSD staff possess low educational attainment, and CSSD team building is not reasonable [21]. Using high-quality professional and technical training to actively promote new ideas, new knowledge, new technology, and new methods to cultivate high-quality CSSD talents is imminent. The training program optimized by action research can effectively develop CSSD talents.

### Limitations of this study

This research is a single-center study, with the study sample limited to nurses from the sterilization supply center of the research unit. The sample size is relatively small, which may introduce a certain degree of bias to the study results. In addition, the limitations of action research should be acknowledged. Examination needs to be conducted at a certain interval after nurse training to check the effectiveness of nurse training. Conducting an examination immediately after completing training has a certain influence. The results are influenced by the memory of participating nurses, their emotions during the examination, etc. We hope to expand the sample size in the future, conduct studies involving multiple units and centers, and continuously engage in a cycle of “Plan-Act-Observe-Reflect” steps to address the needs of the actual situation.

## Conclusions

Action research is widely used in undergraduate education and clinical education for nursing students, including nursing undergraduate curriculum reform, clinical nursing practice teaching, nursing management, etc. It can effectively solve practical problems. The implementation steps of action research are not significantly different from those of general empirical research. However, the main difference between action research and empirical research is the developmental nature of action research (which is also a characteristic of action research). The content of an action research program is not set in stone once it is decided upon, but is allowed and must be reviewed at any time, and continually revised to meet the needs of the actual situation until the research is completed. Thus, this also shows the significance of “action” in action research. This study elucidates the optimization of the training program for the sterilization supply center based on the action research method. The research participants actively engaged in the research process, and, being in a collaborative position with the researcher, continuously raised questions and provided improvement suggestions, aiming to achieve optimal results. The optimized training program not only elevated the professional knowledge and required skills of nurses in the sterilization supply center but also contributed to enhancing their interest in learning. The research findings confirm that the implementation of the optimized training program resulted in significant improvements in nurses’ theoretical and practical performance in areas such as basic knowledge, specialized knowledge, regulations, infection control, institutional culture, party building, and management knowledge compared to the pre-optimization phase. The addition of diverse knowledge types based on nurses’ learning preferences increased their motivation to learn, leading to an overall improvement in satisfaction. The implementation of the training program based on action research establishes a solid foundation for the development of professional talents in the central sterile supply department.

## Data Availability

The data analysed during the study will be made available by the corresponding author upon reasonable request.
